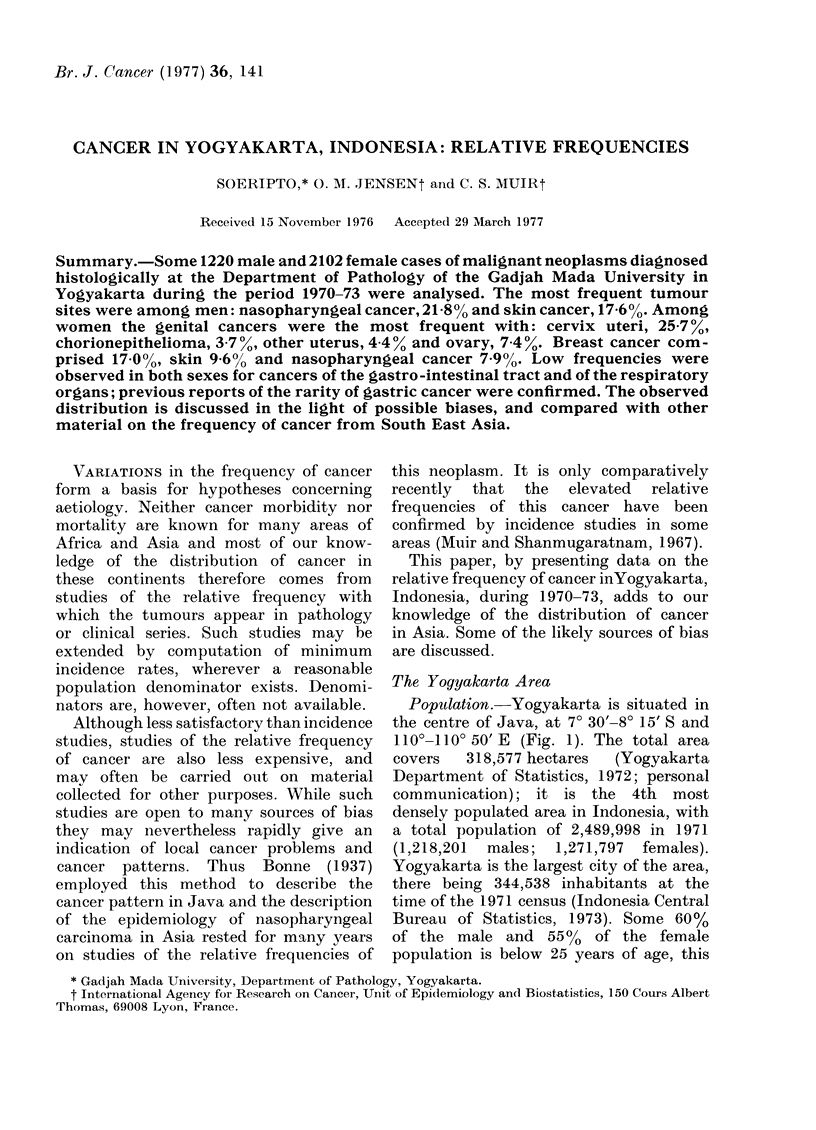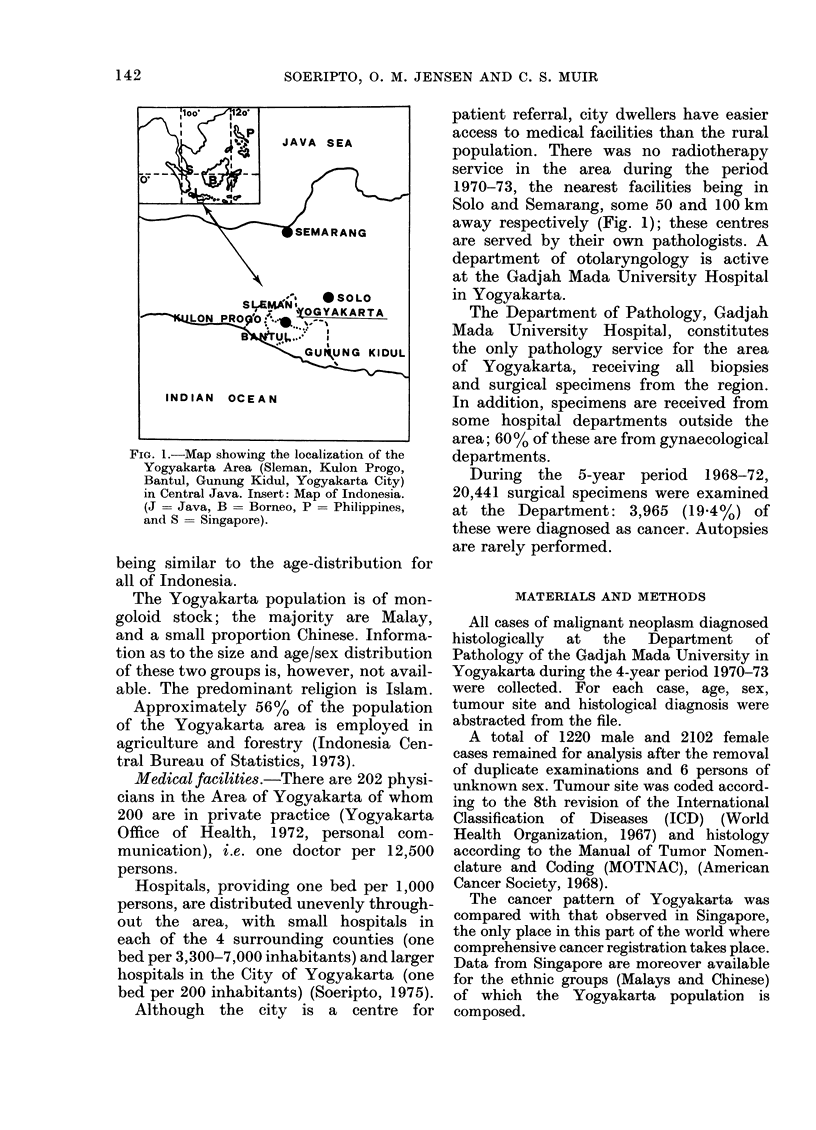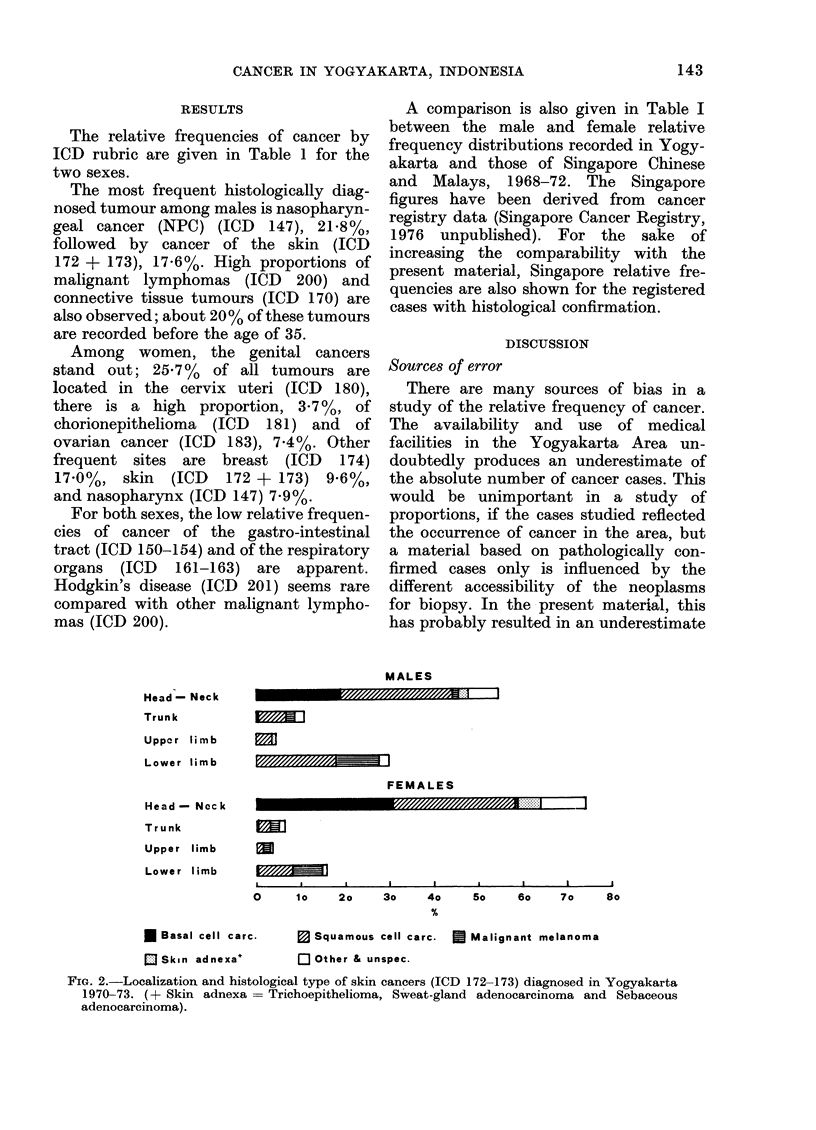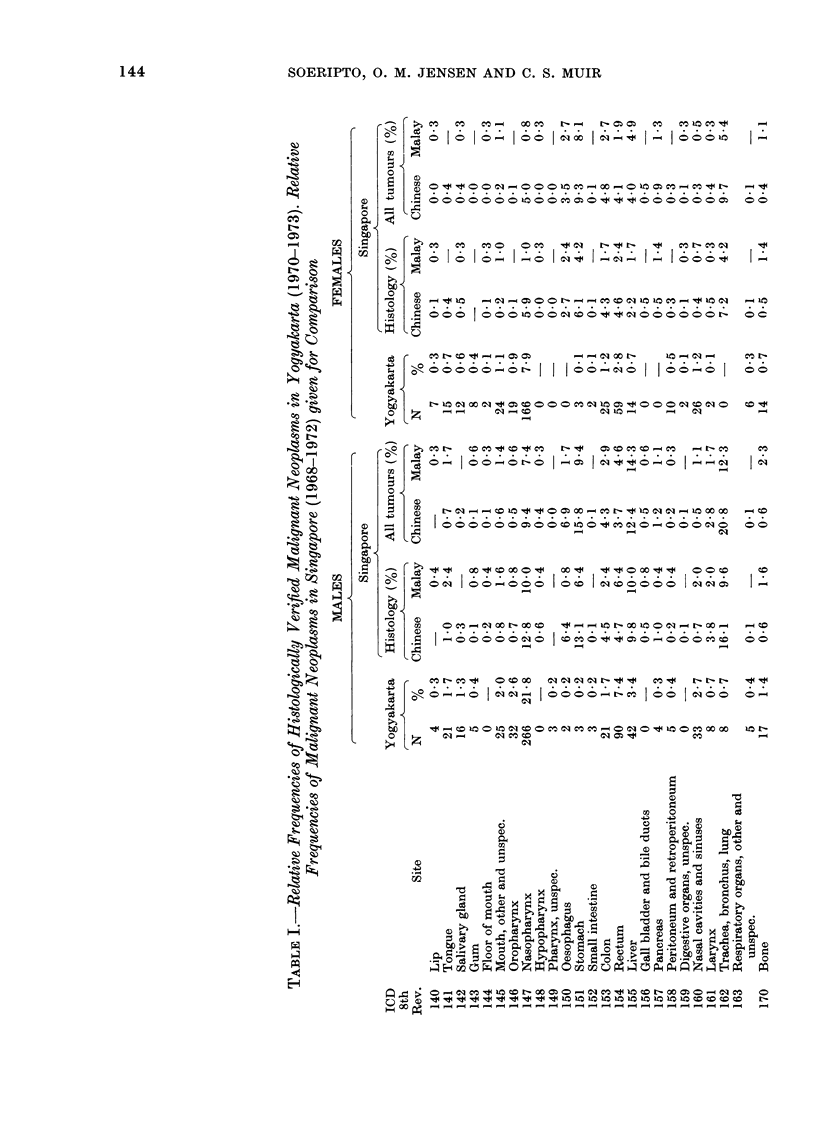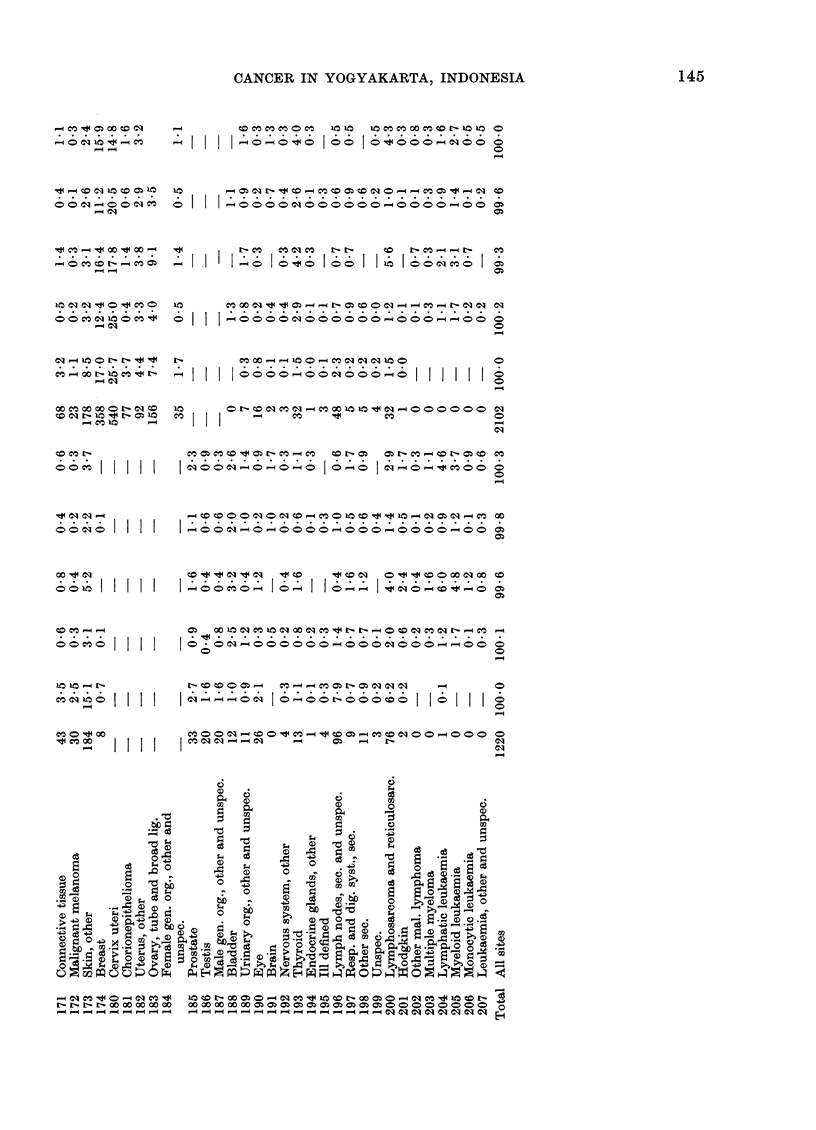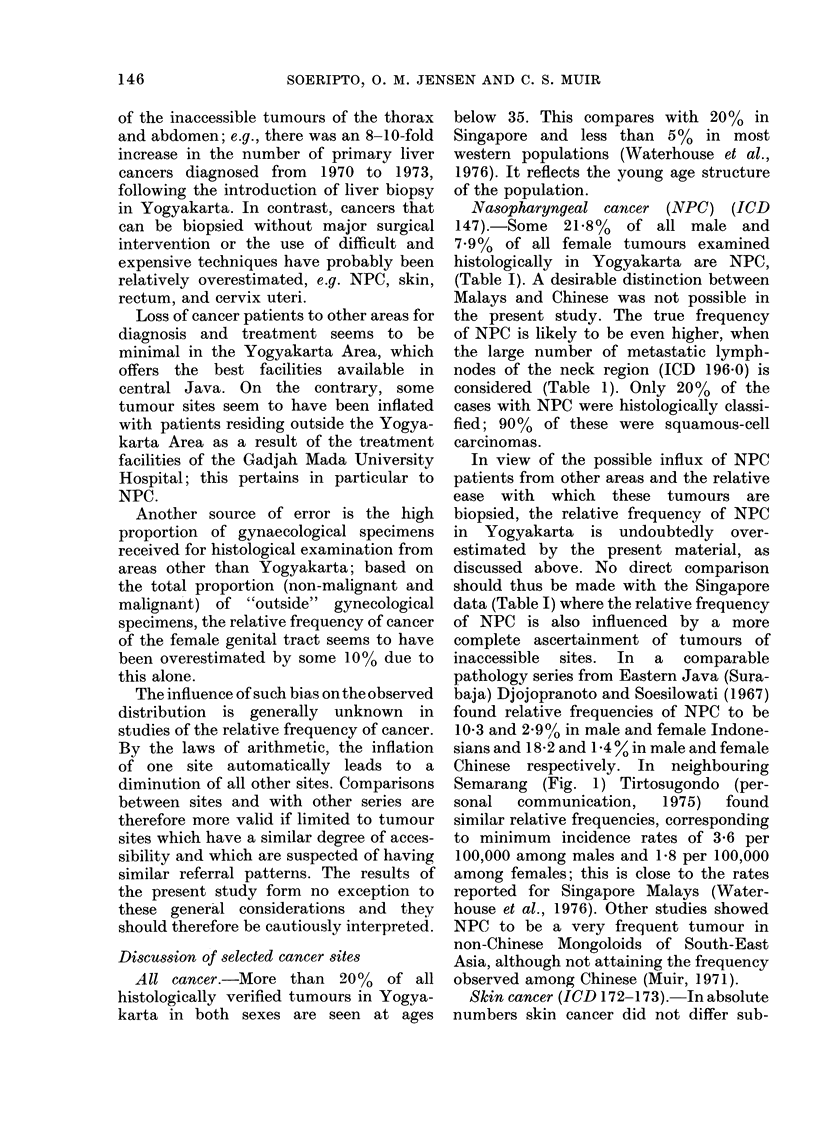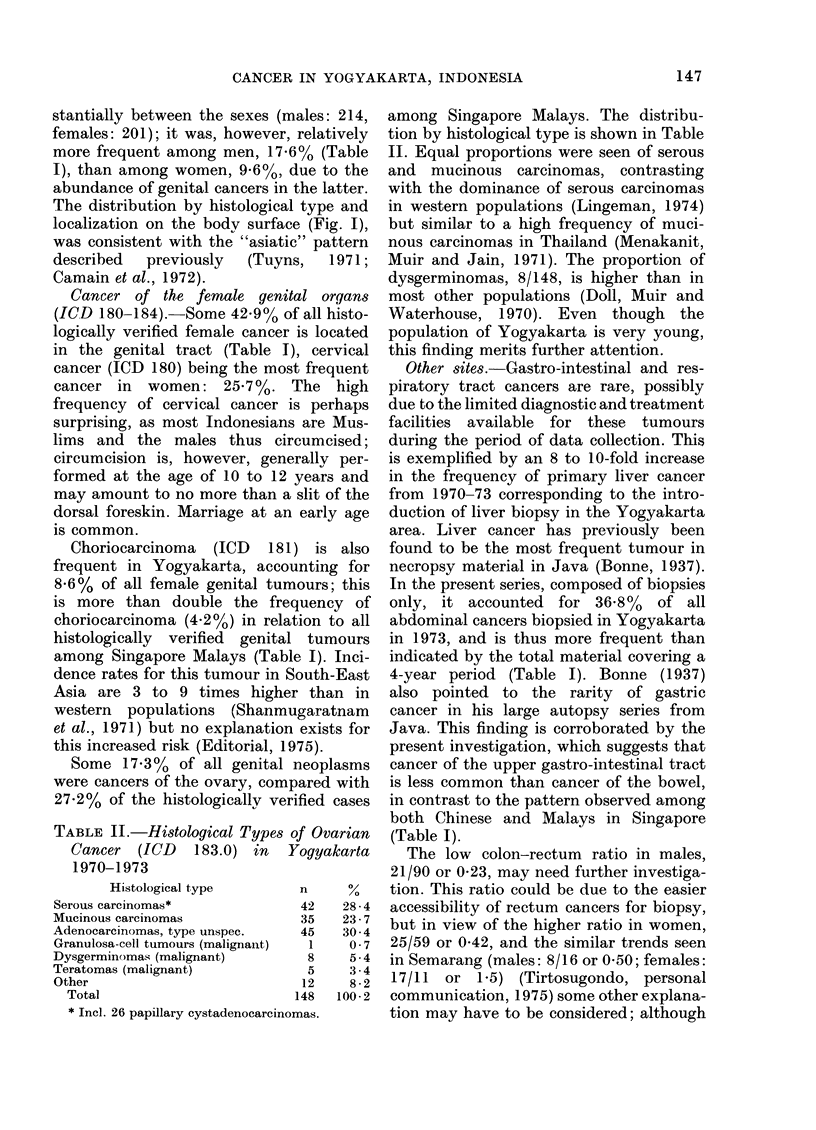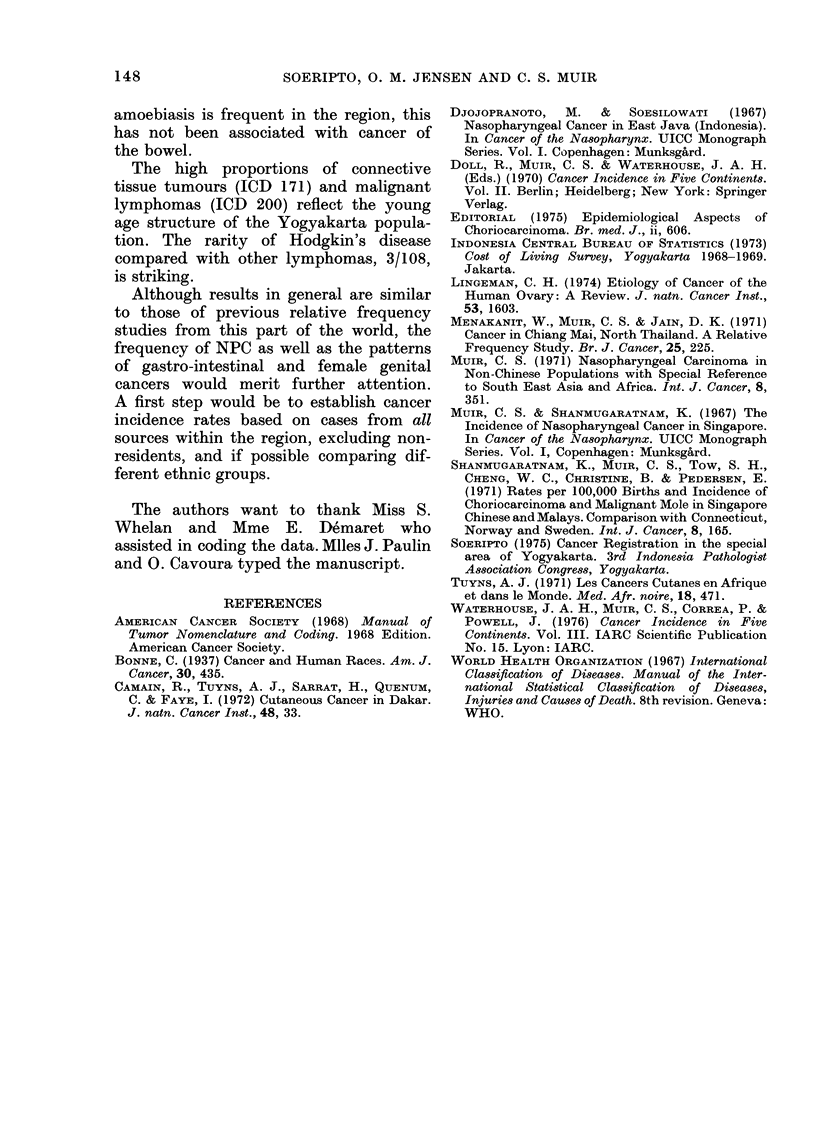# Cancer in Yogyakarta, Indonesia: relative frequencies.

**DOI:** 10.1038/bjc.1977.164

**Published:** 1977-07

**Authors:** O. M. Jensen, C. S. Muir

## Abstract

Some 1220 male and 2102 female cases of malignant neoplasms diagnosed histologically at the Department of Pathology of the Gadjah Mada University in Yogyakarta during the period 1970-73 were analysed. The most frequent tumour sites were among men: nasopharyngeal cancer, 21-8% and skin cancer, 17-6%. Among women the genital cancers were the most frequent with: cervix uteri, 25-7% chorionepithelioma, 3-7%, other uterus, 4-4% and ovary, 7-4%. Breast cancer comprised 17-0%, skin 9-6% and nasopharyngeal cancer 7-9%. Low frequencies were observed in both sexes for cancers of the gastro-intestinal tract and of the respiratory organs; previous reports of the rarity of gastric cancer were confirmed. The observed distribution is discussed in the light of possible biases, and compared with other material on the frequency of cancer from South East Asia.


*Br. J. Cancer* (1977) **36**, 141

### CANCER IN YOGYAKARTA, INDONESIA: RELATIVE FREQUENCIES

SOERIPTO,* O. M. JENSEN† and C. S. MUIR†

Received 15 November 1976 Accepted 29 March 1977

**Summary.—Some 1220 male and 2102 female cases of malignant neoplasms diagnosed histologically at the Department of Pathology of the Gadjah Mada University in Yogyakarta during the period 1970–73 were analysed. The most frequent tumour sites were among men: nasopharyngeal cancer, 21·8% and skin cancer, 17·6%. Among women the genital cancers were the most frequent with: cervix uteri, 25·7%, chorionepithelioma, 3·7%, other uterus, 4·4% and ovary, 7·4%. Breast cancer comprised 17·0%, skin 9·6% and nasopharyngeal cancer 7·9%. Low frequencies were observed in both sexes for cancers of the gastro-intestinal tract and of the respiratory organs; previous reports of the rarity of gastric cancer were confirmed. The observed distribution is discussed in the light of possible biases, and compared with other material on the frequency of cancer from South East Asia.**

VARIATIONS in the frequency of cancer form a basis for hypotheses concerning aetiology. Neither cancer morbidity nor mortality are known for many areas of Africa and Asia and most of our knowledge of the distribution of cancer in these continents therefore comes from studies of the relative frequency with which the tumours appear in pathology or clinical series. Such studies may be extended by computation of minimum incidence rates, wherever a reasonable population denominator exists. Denominators are, however, often not available.

Although less satisfactory than incidence studies, studies of the relative frequency of cancer are also less expensive, and may often be carried out on material collected for other purposes. While such studies are open to many sources of bias they may nevertheless rapidly give an indication of local cancer problems and cancer patterns. Thus Bonne (1937) employed this method to describe the cancer pattern in Java and the description of the epidemiology of nasopharyngeal carcinoma in Asia rested for many years on studies of the relative frequencies of this neoplasm. It is only comparatively recently that the elevated relative frequencies of this cancer have been confirmed by incidence studies in some areas (Muir and Shanmugaratnam, 1967).

This paper, by presenting data on the relative frequency of cancer in Yogyakarta, Indonesia, during 1970–73, adds to our knowledge of the distribution of cancer in Asia. Some of the likely sources of bias are discussed.

### *The Yogyakarta Area*

*Population.*—Yogyakarta is situated in the centre of Java, at 7° 30′–8° 15′ S and 110°–110° 50′ E (Fig. 1). The total area covers 318,577 hectares (Yogyakarta Department of Statistics, 1972; personal communication); it is the 4th most densely populated area in Indonesia, with a total population of 2,489,998 in 1971 (1,218,201 males; 1,271,797 females). Yogyakarta is the largest city of the area, there being 344,538 inhabitants at the time of the 1971 census (Indonesia Central Bureau of Statistics, 1973). Some 60% of the male and 55% of the female population is below 25 years of age, this

* Gadjah Mada University, Department of Pathology, Yogyakarta.

† International Agency for Research on Cancer, Unit of Epidemiology and Biostatistics, 150 Cours Albert Thomas, 69008 Lyon, France.

FIG. 1.—Map showing the localization of the Yogyakarta Area (Sleman, Kulon Progo, Bantul, Gunung Kidul, Yogyakarta City) in Central Java. Insert: Map of Indonesia. (J = Java, B = Borneo, P = Philippines, and S = Singapore).

being similar to the age-distribution for all of Indonesia.

The Yogyakarta population is of mongoloid stock; the majority are Malay, and a small proportion Chinese. Information as to the size and age/sex distribution of these two groups is, however, not available. The predominant religion is Islam.

Approximately 56% of the population of the Yogyakarta area is employed in agriculture and forestry (Indonesia Central Bureau of Statistics, 1973).

*Medical facilities*.—There are 202 physicians in the Area of Yogyakarta of whom 200 are in private practice (Yogyakarta Office of Health, 1972, personal communication), *i.e.* one doctor per 12,500 persons.

Hospitals, providing one bed per 1,000 persons, are distributed unevenly throughout the area, with small hospitals in each of the 4 surrounding counties (one bed per 3,300–7,000 inhabitants) and larger hospitals in the City of Yogyakarta (one bed per 200 inhabitants) (Soeripto, 1975).

Although the city is a centre for patient referral, city dwellers have easier access to medical facilities than the rural population. There was no radiotherapy service in the area during the period 1970–73, the nearest facilities being in Solo and Semarang, some 50 and 100 km away respectively (Fig. 1); these centres are served by their own pathologists. A department of otolaryngology is active at the Gadjah Mada University Hospital in Yogyakarta.

The Department of Pathology, Gadjah Mada University Hospital, constitutes the only pathology service for the area of Yogyakarta, receiving all biopsies and surgical specimens from the region. In addition, specimens are received from some hospital departments outside the area; 60% of these are from gynaecological departments.

During the 5-year period 1968–72, 20,441 surgical specimens were examined at the Department: 3,965 (19·4%) of these were diagnosed as cancer. Autopsies are rarely performed.

### MATERIALS AND METHODS

All cases of malignant neoplasm diagnosed histologically at the Department of Pathology of the Gadjah Mada University in Yogyakarta during the 4-year period 1970–73 were collected. For each case, age, sex, tumour site and histological diagnosis were abstracted from the file.

A total of 1220 male and 2102 female cases remained for analysis after the removal of duplicate examinations and 6 persons of unknown sex. Tumour site was coded according to the 8th revision of the International Classification of Diseases (ICD) (World Health Organization, 1967) and histology according to the Manual of Tumor Nomenclature and Coding (MOTNAC), (American Cancer Society, 1968).

The cancer pattern of Yogyakarta was compared with that observed in Singapore, the only place in this part of the world where comprehensive cancer registration takes place. Data from Singapore are moreover available for the ethnic groups (Malays and Chinese) of which the Yogyakarta population is composed.

RESULTS

The relative frequencies of cancer by ICD rubric are given in Table 1 for the two sexes.

The most frequent histologically diagnosed tumour among males is nasopharyngeal cancer (NPC) (ICD 147), 21·8%, followed by cancer of the skin (ICD 172 + 173), 17·6%. High proportions of malignant lymphomas (ICD 200) and connective tissue tumours (ICD 170) are also observed; about 20% of these tumours are recorded before the age of 35.

Among women, the genital cancers stand out; 25·7% of all tumours are located in the cervix uteri (ICD 180), there is a high proportion, 3·7%, of chorionepithelioma (ICD 181) and of ovarian cancer (ICD 183), 7·4%. Other frequent sites are breast (ICD 174) 17·0%, skin (ICD 172 + 173) 9·6%, and nasopharynx (ICD 147) 7·9%.

For both sexes, the low relative frequencies of cancer of the gastro-intestinal tract (ICD 150–154) and of the respiratory organs (ICD 161–163) are apparent. Hodgkin's disease (ICD 201) seems rare compared with other malignant lymphomas (ICD 200).

A comparison is also given in Table I between the male and female relative frequency distributions recorded in Yogyakarta and those of Singapore Chinese and Malays, 1968–72. The Singapore figures have been derived from cancer registry data (Singapore Cancer Registry, 1976 unpublished). For the sake of increasing the comparability with the present material, Singapore relative frequencies are also shown for the registered cases with histological confirmation.

DISCUSSION

*Sources of error*

There are many sources of bias in a study of the relative frequency of cancer. The availability and use of medical facilities in the Yogyakarta Area undoubtedly produces an underestimate of the absolute number of cancer cases. This would be unimportant in a study of proportions, if the cases studied reflected the occurrence of cancer in the area, but a material based on pathologically confirmed cases only is influenced by the different accessibility of the neoplasms for biopsy. In the present material, this has probably resulted in an underestimate

FIG. 2.—Localization and histological type of skin cancers (ICD 172–173) diagnosed in Yogyakarta 1970–73. (+ Skin adnexa = Trichoepithelioma, Sweat-gland adenocarcinoma and Sebaceous adenocarcinoma).

TABLE I.—*Relative Frequencies of Histologically Verified Malignant Neoplasms in Yogyakarta* (1970–1973). *Relative Frequencies of Malignant Neoplasms in Singapore* (1968–1972) *given for Comparison*

| ICD 8th Rev. | Site | MALES: Yogyakarta N | MALES: Yogyakarta % | MALES: Singapore Histology (%) Chinese | MALES: Singapore Histology (%) Malay | MALES: Singapore All tumours (%) Chinese | MALES: Singapore All tumours (%) Malay | FEMALES: Yogyakarta N | FEMALES: Yogyakarta % | FEMALES: Singapore Histology (%) Chinese | FEMALES: Singapore Histology (%) Malay | FEMALES: Singapore All tumours (%) Chinese | FEMALES: Singapore All tumours (%) Malay |
|---|---|---|---|---|---|---|---|---|---|---|---|---|---|
| 140 | Lip | 4 | 0·3 | — | 0·4 | — | 0·3 | 7 | 0·3 | 0·1 | 0·3 | 0·0 | 0·3 |
| 141 | Tongue | 21 | 1·7 | 1·0 | 2·4 | 0·7 | 1·7 | 15 | 0·7 | 0·4 | — | 0·4 | — |
| 142 | Salivary gland | 16 | 1·3 | 0·3 | — | 0·2 | — | 12 | 0·6 | 0·5 | 0·3 | 0·4 | 0·3 |
| 143 | Gum | 5 | 0·4 | 0·1 | 0·8 | 0·1 | 0·6 | 8 | 0·4 | — | — | 0·0 | — |
| 144 | Floor of mouth | 0 | — | 0·2 | 0·4 | 0·1 | 0·3 | 2 | 0·1 | 0·1 | 0·3 | 0·0 | 0·3 |
| 145 | Mouth, other and unspec. | 25 | 2·0 | 0·8 | 1·6 | 0·6 | 1·4 | 24 | 1·1 | 0·2 | 1·0 | 0·2 | 1·1 |
| 146 | Oropharynx | 32 | 2·6 | 0·7 | 0·8 | 0·5 | 0·6 | 19 | 0·9 | 0·1 | — | 0·1 | — |
| 147 | Nasopharynx | 266 | 21·8 | 12·8 | 10·0 | 9·4 | 7·4 | 166 | 7·9 | 5·9 | 1·0 | 5·0 | 0·8 |
| 148 | Hypopharynx | 0 | — | 0·6 | 0·4 | 0·4 | 0·3 | 0 | — | 0·0 | 0·3 | 0·0 | 0·3 |
| 149 | Pharynx, unspec. | 3 | 0·2 | — | — | 0·0 | — | 0 | — | 0·0 | — | 0·0 | — |
| 150 | Oesophagus | 2 | 0·2 | 6·4 | 0·8 | 6·9 | 1·7 | 0 | — | 2·7 | 2·4 | 3·5 | 2·7 |
| 151 | Stomach | 3 | 0·2 | 13·1 | 6·4 | 15·8 | 9·4 | 3 | 0·1 | 6·1 | 4·2 | 9·3 | 8·1 |
| 152 | Small intestine | 3 | 0·2 | 0·1 | — | 0·1 | — | 2 | 0·1 | 0·1 | — | 0·1 | — |
| 153 | Colon | 21 | 1·7 | 4·5 | 2·4 | 4·3 | 2·9 | 25 | 1·2 | 4·3 | 1·7 | 4·8 | 2·7 |
| 154 | Rectum | 90 | 7·4 | 4·7 | 6·4 | 3·7 | 4·6 | 59 | 2·8 | 4·6 | 2·4 | 4·1 | 1·9 |
| 155 | Liver | 42 | 3·4 | 9·8 | 10·0 | 12·4 | 14·3 | 14 | 0·7 | 2·2 | 1·7 | 4·0 | 4·9 |
| 156 | Gall bladder and bile ducts | 0 | — | 0·5 | 0·8 | 0·5 | 0·6 | 0 | — | 0·5 | — | 0·5 | — |
| 157 | Pancreas | 4 | 0·3 | 1·0 | 0·4 | 1·2 | 1·1 | 0 | — | 0·5 | 1·4 | 0·9 | 1·3 |
| 158 | Peritoneum and retroperitoneum | 5 | 0·4 | 0·2 | 0·4 | 0·2 | 0·3 | 10 | 0·5 | 0·3 | — | 0·3 | — |
| 159 | Digestive organs, unspec. | 0 | — | 0·1 | — | 0·1 | — | 2 | 0·1 | 0·1 | 0·3 | 0·1 | 0·3 |
| 160 | Nasal cavities and sinuses | 33 | 2·7 | 0·7 | 2·0 | 0·5 | 1·1 | 26 | 1·2 | 0·4 | 0·7 | 0·3 | 0·5 |
| 161 | Larynx | 8 | 0·7 | 3·8 | 2·0 | 2·8 | 1·7 | 2 | 0·1 | 0·5 | 0·3 | 0·4 | 0·3 |
| 162 | Trachea, bronchus, lung | 8 | 0·7 | 16·1 | 9·6 | 20·8 | 12·3 | 0 | — | 7·2 | 4·2 | 9·7 | 5·4 |
| 163 | Respiratory organs, other and unspec. | 5 | 0·4 | 0·1 | — | 0·1 | — | 6 | 0·3 | 0·1 | — | 0·1 | — |
| 170 | Bone | 17 | 1·4 | 0·6 | 1·6 | 0·6 | 2·3 | 14 | 0·7 | 0·5 | 1·4 | 0·4 | 1·1 |

| | | | | | | | | | | | | | |
|---|---|---|---|---|---|---|---|---|---|---|---|---|---|
| 171 | Connective tissue | 43 | 3·5 | 0·6 | 0·8 | 0·4 | 0·6 | 68 | 3·2 | 0·5 | 1·4 | 0·4 | 1·1 |
| 172 | Malignant melanoma | 30 | 2·5 | 0·3 | 0·4 | 0·2 | 0·3 | 23 | 1·1 | 0·2 | 0·3 | 0·1 | 0·3 |
| 173 | Skin, other | 184 | 15·1 | 3·1 | 5·2 | 2·2 | 3·7 | 178 | 8·5 | 3·2 | 3·1 | 2·6 | 2·4 |
| 174 | Breast | 8 | 0·7 | 0·1 | — | 0·1 | — | 358 | 17·0 | 12·4 | 16·4 | 11·2 | 15·9 |
| 180 | Cervix uteri | — | — | — | — | — | — | 540 | 25·7 | 25·0 | 17·8 | 20·5 | 14·8 |
| 181 | Chorionepithelioma | — | — | — | — | — | — | 77 | 3·7 | 0·4 | 1·4 | 0·6 | 1·6 |
| 182 | Uterus, other | — | — | — | — | — | — | 92 | 4·4 | 3·3 | 3·8 | 2·9 | 3·2 |
| 183 | Ovary, tube and broad lig. | — | — | — | — | — | — | 156 | 7·4 | 4·0 | 9·1 | 3·5 | |
| 184 | Female gen. org., other and unspec. | — | — | — | — | — | — | 35 | 1·7 | 0·5 | 1·4 | 0·5 | 1·1 |
| 185 | Prostate | 33 | 2·7 | 0·9 | 1·6 | 1·1 | 2·3 | — | — | — | — | — | — |
| 186 | Testis | 20 | 1·6 | 0·4 | 0·4 | 0·6 | 0·9 | — | — | — | — | — | — |
| 187 | Male gen. org., other and unspec. | 20 | 1·6 | 0·8 | 0·4 | 0·6 | 0·3 | — | — | — | — | — | — |
| 188 | Bladder | 12 | 1·0 | 2·5 | 3·2 | 2·0 | 2·6 | 0 | — | 1·3 | — | 1·1 | — |
| 189 | Urinary org., other and unspec. | 11 | 0·9 | 1·2 | 0·4 | 1·0 | 1·4 | 7 | 0·3 | 0·8 | 1·7 | 0·9 | 1·6 |
| 190 | Eye | 26 | 2·1 | 0·3 | 1·2 | 0·2 | 0·9 | 16 | 0·8 | 0·2 | 0·3 | 0·2 | 0·3 |
| 191 | Brain | 0 | — | 0·5 | — | 1·0 | 1·7 | 2 | 0·1 | 0·4 | — | 0·7 | 1·3 |
| 192 | Nervous system, other | 4 | 0·3 | 0·2 | 0·4 | 0·2 | 0·3 | 3 | 0·1 | 0·4 | 0·3 | 0·4 | 0·3 |
| 193 | Thyroid | 13 | 1·1 | 0·8 | 1·6 | 0·6 | 1·1 | 32 | 1·5 | 2·9 | 4·2 | 2·6 | 4·0 |
| 194 | Endocrine glands, other | 1 | 0·1 | 0·2 | — | 0·1 | 0·3 | 1 | 0·0 | 0·1 | 0·3 | 0·1 | 0·3 |
| 195 | Ill defined | 4 | 0·3 | 0·3 | — | 0·3 | — | 3 | 0·1 | 0·1 | — | 0·3 | — |
| 196 | Lymph nodes, sec. and unspec. | 96 | 7·9 | 1·4 | 0·4 | 1·0 | 0·6 | 48 | 2·3 | 0·7 | 0·7 | 0·6 | 0·5 |
| 197 | Resp. and dig. syst., sec. | 9 | 0·7 | 0·7 | 1·6 | 0·5 | 1·7 | 5 | 0·2 | 0·9 | 0·7 | 0·9 | 0·5 |
| 198 | Other sec. | 11 | 0·9 | 0·7 | 1·2 | 0·6 | 0·9 | 5 | 0·2 | 0·6 | — | 0·6 | — |
| 199 | Unspec. | 3 | 0·2 | 0·1 | — | 0·4 | — | 4 | 0·2 | 0·0 | — | 0·2 | 0·5 |
| 200 | Lymphosarcoma and reticulosarc. | 76 | 6·2 | 2·0 | 4·0 | 1·4 | 2·9 | 32 | 1·5 | 1·2 | 5·6 | 1·0 | 4·3 |
| 201 | Hodgkin | 2 | 0·2 | 0·6 | 2·4 | 0·5 | 1·7 | 1 | 0·0 | 0·1 | — | 0·1 | 0·3 |
| 202 | Other mal. lymphoma | 0 | — | 0·2 | 0·4 | 0·1 | 0·3 | 0 | — | 0·1 | 0·7 | 0·1 | 0·8 |
| 203 | Multiple myeloma | 0 | — | 0·3 | 1·6 | 0·2 | 1·1 | 0 | — | 0·3 | 0·3 | 0·3 | 0·3 |
| 204 | Lymphatic leukaemia | 1 | 0·1 | 1·2 | 6·0 | 0·9 | 4·6 | 0 | — | 1·1 | 2·1 | 0·9 | 1·6 |
| 205 | Myeloid leukaemia | 0 | — | 1·7 | 4·8 | 1·2 | 3·7 | 0 | — | 1·7 | 3·1 | 1·4 | 2·7 |
| 206 | Monocytic leukaemia | 0 | — | 0·1 | 1·2 | 0·1 | 0·9 | 0 | — | 0·2 | 0·7 | 0·1 | 0·5 |
| 207 | Leukaemia, other and unspec. | 0 | — | 0·3 | 0·8 | 0·3 | 0·6 | 0 | — | 0·2 | — | 0·2 | 0·5 |
| Total | All sites | 1220 | 100·0 | 100·1 | 99·6 | 99·8 | 100·3 | 2102 | 100·0 | 100·2 | 99·3 | 99·6 | 100·0 |

of the inaccessible tumours of the thorax and abdomen; *e.g.*, there was an 8–10-fold increase in the number of primary liver cancers diagnosed from 1970 to 1973, following the introduction of liver biopsy in Yogyakarta. In contrast, cancers that can be biopsied without major surgical intervention or the use of difficult and expensive techniques have probably been relatively overestimated, *e.g.* NPC, skin, rectum, and cervix uteri.

Loss of cancer patients to other areas for diagnosis and treatment seems to be minimal in the Yogyakarta Area, which offers the best facilities available in central Java. On the contrary, some tumour sites seem to have been inflated with patients residing outside the Yogyakarta Area as a result of the treatment facilities of the Gadjah Mada University Hospital; this pertains in particular to NPC.

Another source of error is the high proportion of gynaecological specimens received for histological examination from areas other than Yogyakarta; based on the total proportion (non-malignant and malignant) of "outside" gynecological specimens, the relative frequency of cancer of the female genital tract seems to have been overestimated by some 10% due to this alone.

The influence of such bias on the observed distribution is generally unknown in studies of the relative frequency of cancer. By the laws of arithmetic, the inflation of one site automatically leads to a diminution of all other sites. Comparisons between sites and with other series are therefore more valid if limited to tumour sites which have a similar degree of accessibility and which are suspected of having similar referral patterns. The results of the present study form no exception to these general considerations and they should therefore be cautiously interpreted.

*Discussion of selected cancer sites*

*All cancer.*—More than 20% of all histologically verified tumours in Yogyakarta in both sexes are seen at ages below 35. This compares with 20% in Singapore and less than 5% in most western populations (Waterhouse *et al.*, 1976). It reflects the young age structure of the population.

*Nasopharyngeal cancer* (*NPC*) (*ICD* 147).—Some 21·8% of all male and 7·9% of all female tumours examined histologically in Yogyakarta are NPC, (Table I). A desirable distinction between Malays and Chinese was not possible in the present study. The true frequency of NPC is likely to be even higher, when the large number of metastatic lymph-nodes of the neck region (ICD 196·0) is considered (Table 1). Only 20% of the cases with NPC were histologically classified; 90% of these were squamous-cell carcinomas.

In view of the possible influx of NPC patients from other areas and the relative ease with which these tumours are biopsied, the relative frequency of NPC in Yogyakarta is undoubtedly overestimated by the present material, as discussed above. No direct comparison should thus be made with the Singapore data (Table I) where the relative frequency of NPC is also influenced by a more complete ascertainment of tumours of inaccessible sites. In a comparable pathology series from Eastern Java (Surabaja) Djojopranoto and Soesilowati (1967) found relative frequencies of NPC to be 10·3 and 2·9% in male and female Indonesians and 18·2 and 1·4% in male and female Chinese respectively. In neighbouring Semarang (Fig. 1) Tirtosugondo (personal communication, 1975) found similar relative frequencies, corresponding to minimum incidence rates of 3·6 per 100,000 among males and 1·8 per 100,000 among females; this is close to the rates reported for Singapore Malays (Waterhouse *et al.*, 1976). Other studies showed NPC to be a very frequent tumour in non-Chinese Mongoloids of South-East Asia, although not attaining the frequency observed among Chinese (Muir, 1971).

*Skin cancer* (*ICD* 172–173).—In absolute numbers skin cancer did not differ substantially between the sexes (males: 214, females: 201); it was, however, relatively more frequent among men, 17·6% (Table I), than among women, 9·6%, due to the abundance of genital cancers in the latter. The distribution by histological type and localization on the body surface (Fig. I), was consistent with the "asiatic" pattern described previously (Tuyns, 1971; Camain *et al.*, 1972).

*Cancer of the female genital organs* (*ICD* 180–184).—Some 42·9% of all histologically verified female cancer is located in the genital tract (Table I), cervical cancer (ICD 180) being the most frequent cancer in women: 25·7%. The high frequency of cervical cancer is perhaps surprising, as most Indonesians are Muslims and the males thus circumcised; circumcision is, however, generally performed at the age of 10 to 12 years and may amount to no more than a slit of the dorsal foreskin. Marriage at an early age is common.

Choriocarcinoma (ICD 181) is also frequent in Yogyakarta, accounting for 8·6% of all female genital tumours; this is more than double the frequency of choriocarcinoma (4·2%) in relation to all histologically verified genital tumours among Singapore Malays (Table I). Incidence rates for this tumour in South-East Asia are 3 to 9 times higher than in western populations (Shanmugaratnam *et al.*, 1971) but no explanation exists for this increased risk (Editorial, 1975).

Some 17·3% of all genital neoplasms were cancers of the ovary, compared with 27·2% of the histologically verified cases among Singapore Malays. The distribution by histological type is shown in Table II. Equal proportions were seen of serous and mucinous carcinomas, contrasting with the dominance of serous carcinomas in western populations (Lingeman, 1974) but similar to a high frequency of mucinous carcinomas in Thailand (Menakanit, Muir and Jain, 1971). The proportion of dysgerminomas, 8/148, is higher than in most other populations (Doll, Muir and Waterhouse, 1970). Even though the population of Yogyakarta is very young, this finding merits further attention.

TABLE II.—*Histological Types of Ovarian Cancer* (*ICD* 183.0) *in Yogyakarta* 1970–1973

| Histological type | n | % |
|---|---|---|
| Serous carcinomas* | 42 | 28·4 |
| Mucinous carcinomas | 35 | 23·7 |
| Adenocarcinomas, type unspec. | 45 | 30·4 |
| Granulosa-cell tumours (malignant) | 1 | 0·7 |
| Dysgerminomas (malignant) | 8 | 5·4 |
| Teratomas (malignant) | 5 | 3·4 |
| Other | 12 | 8·2 |
| Total | 148 | 100·2 |

* Incl. 26 papillary cystadenocarcinomas.

*Other sites.*—Gastro-intestinal and respiratory tract cancers are rare, possibly due to the limited diagnostic and treatment facilities available for these tumours during the period of data collection. This is exemplified by an 8 to 10-fold increase in the frequency of primary liver cancer from 1970–73 corresponding to the introduction of liver biopsy in the Yogyakarta area. Liver cancer has previously been found to be the most frequent tumour in necropsy material in Java (Bonne, 1937). In the present series, composed of biopsies only, it accounted for 36·8% of all abdominal cancers biopsied in Yogyakarta in 1973, and is thus more frequent than indicated by the total material covering a 4-year period (Table I). Bonne (1937) also pointed to the rarity of gastric cancer in his large autopsy series from Java. This finding is corroborated by the present investigation, which suggests that cancer of the upper gastro-intestinal tract is less common than cancer of the bowel, in contrast to the pattern observed among both Chinese and Malays in Singapore (Table I).

The low colon–rectum ratio in males, 21/90 or 0·23, may need further investigation. This ratio could be due to the easier accessibility of rectum cancers for biopsy, but in view of the higher ratio in women, 25/59 or 0·42, and the similar trends seen in Semarang (males: 8/16 or 0·50; females: 17/11 or 1·5) (Tirtosugondo, personal communication, 1975) some other explanation may have to be considered; although amoebiasis is frequent in the region, this has not been associated with cancer of the bowel.

The high proportions of connective tissue tumours (ICD 171) and malignant lymphomas (ICD 200) reflect the young age structure of the Yogyakarta population. The rarity of Hodgkin's disease compared with other lymphomas, 3/108, is striking.

Although results in general are similar to those of previous relative frequency studies from this part of the world, the frequency of NPC as well as the patterns of gastro-intestinal and female genital cancers would merit further attention. A first step would be to establish cancer incidence rates based on cases from *all* sources within the region, excluding non-residents, and if possible comparing different ethnic groups.

The authors want to thank Miss S. Whelan and Mme E. Démaret who assisted in coding the data. Mlles J. Paulin and O. Cavoura typed the manuscript.

### REFERENCES

American Cancer Society (1968) *Manual of Tumor Nomenclature and Coding.* 1968 Edition. American Cancer Society.

Bonne, C. (1937) Cancer and Human Races. *Am. J. Cancer*, **30**, 435.

Camain, R., Tuyns, A. J., Sarrat, H., Quenum, C. & Faye, I. (1972) Cutaneous Cancer in Dakar. *J. natn. Cancer Inst.*, **48**, 33.

Djojopranoto, M. & Soesilowati (1967) Nasopharyngeal Cancer in East Java (Indonesia). In *Cancer of the Nasopharynx.* UICC Monograph Series. Vol. I. Copenhagen: Munksgård.

Doll, R., Muir, C. S. & Waterhouse, J. A. H. (Eds.) (1970) *Cancer Incidence in Five Continents.* Vol. II. Berlin; Heidelberg; New York: Springer Verlag.

Editorial (1975) Epidemiological Aspects of Choriocarcinoma. *Br. med. J.*, ii, 606.

Indonesia Central Bureau of Statistics (1973) *Cost of Living Survey, Yogyakarta* 1968–1969. Jakarta.

Lingeman, C. H. (1974) Etiology of Cancer of the Human Ovary: A Review. *J. natn. Cancer Inst.*, **53**, 1603.

Menakanit, W., Muir, C. S. & Jain, D. K. (1971) Cancer in Chiang Mai, North Thailand. A Relative Frequency Study. *Br. J. Cancer*, **25**, 225.

Muir, C. S. (1971) Nasopharyngeal Carcinoma in Non-Chinese Populations with Special Reference to South East Asia and Africa. *Int. J. Cancer*, **8**, 351.

Muir, C. S. & Shanmugaratnam, K. (1967) The Incidence of Nasopharyngeal Cancer in Singapore. In *Cancer of the Nasopharynx.* UICC Monograph Series. Vol. I, Copenhagen: Munksgård.

Shanmugaratnam, K., Muir, C. S., Tow, S. H., Cheng, W. C., Christine, B. & Pedersen, E. (1971) Rates per 100,000 Births and Incidence of Choriocarcinoma and Malignant Mole in Singapore Chinese and Malays. Comparison with Connecticut, Norway and Sweden. *Int. J. Cancer*, **8**, 165.

Soeripto (1975) Cancer Registration in the special area of Yogyakarta. *3rd Indonesia Pathologist Association Congress, Yogyakarta.*

Tuyns, A. J. (1971) Les Cancers Cutanes en Afrique et dans le Monde. *Med. Afr. noire*, **18**, 471.

Waterhouse, J. A. H., Muir, C. S., Correa, P. & Powell, J. (1976) *Cancer Incidence in Five Continents.* Vol. III. IARC Scientific Publication No. 15. Lyon: IARC.

World Health Organization (1967) *International Classification of Diseases. Manual of the International Statistical Classification of Diseases, Injuries and Causes of Death.* 8th revision. Geneva: WHO.